# Womens Rights

**Published:** 1869-07

**Authors:** 


### WOMENS RIGHTS.

Taking it for granted, that those ladies who advocate the right of women to vote, at so much cost of time, money, and personal labor, are nobly "contending for the right," as it seems to them, let us look at the question ourselves and let our own consciousness decide whether it is expedient to give women the same right to vote which men have.

Rights and privileges involve corresponding duties and responsibilities. If women vote they should, like men, be subject to jury and military duty, to work on the roads, to answer promptly the summons of the sheriff, to aid in keeping the peace and arresting lawbreakers, thieves and murderers. It would not be seemly for a woman to be thus engaged; everybody must see the inappropriateness of such positions; is there a brother, or son or father, who would not be shocked to find a sister, or mother, or wife thus employed? Is there one lady of refinement in ten thousand who would not instinctively withdraw herself from such a position?

The family relation is at the very foundation of civilized society, it was ordered for the express purpose of happiness and perpetuating the race; and it seems to be the natural business of a woman to manage the household, and that of the man to provide for it. It is the mother who is to rear the child; it is the mother who forms its character, and upon that character the destinies of society and the whole country depend; it is the mother who watches over the child with ineffable tenderness; no other human being is as capable of doing it as she, and any woman who keeps her house as it ought to be kept, and who gives her children proper attention, has quite as much to do as she ought to do. To vote intelligently requires study, inquiry, investigation and deliberate thought, but already the wretched housekeepers of our time will tell you that they have "so much to do"; more to do than allows them to attend the prayer meeting; to visit a poor, sick neighbor now and then; to take him some little delicacy and spend a few moments in encouraging conversation; to visit the widows and the fatherless in their affliction, which is said to be true religion, as to our active duties towards others; to call in your carriage at the humble cottage and take out its weak, worn occupant for a half hour's ride; nine mothers out of ten will tell you they have not the time to do these things, and that their first duties are to their families, but by giving the women the right to vote, a considerable portion of time must be given in connection with the duties and responsibilities of voting. We certainly do not remember to have found in the course of a life time, a single tidy housekeeper who did not complain of having more to do than she could comfortably get along with. There are men, gentlemen, in our large cities who do not go to vote, because they are unwilling to come into proximity with those who ordinarily "crowd the polls"; and if men instinctively shun the defilement, much more will women of refinement do likewise; then the practical result would be to give the votes to the more degraded classes when already our country seems at times to be on the verge of ruin through the already too extended suffrage.

If women could vote there would be another element of domestic discord; in uncounted instances they would be compelled to vote as their husbands did, to vote against their own convictions, and it is as certain as any untranspired event, that many a woman would be brutally beaten and even murdered by ignorant and degraded and drunken husbands, for voting contrary to their wishes. These are not offered as original arguments, but they are some of the considerations which have inclined us to the belief, if women are allowed to vote, their own degradation would be a direct and an inevitable result; it would leave them less time for the management of their households; their children would be more neglected; their bodies less cared for and thus become more exposed to accidents and disease while their affections and their intellects, being under less training than now, would be perverted and the whole structure of society would be shattered if not wholly broken up. Woman is in her highest glory at the fireside, at the head of the table and in the drawing room; on the platform or at the polls that glory fades away, and there is no resurrection; for we remember no case in all history where a woman who had justly lost public appreciation, ever recovered it again.

